# HOSPITAL ADMISSIONS OF ADOLESCENTS IN SERGIPE, FROM 2002 TO 2012

**DOI:** 10.1590/1984-0462/2020/38/2018181

**Published:** 2019-11-25

**Authors:** Nynemberg Menezes Guimarães, Eleonora Ramos de Oliveira, Anna Klara Bohland

**Affiliations:** a Department of Medicine, Universidade Federal de Sergipe, Aracaju, SE, Brazil.; b Department of Medicine, Universidade Federal da Paraíba, João Pessoa, PB, Brazil.

**Keywords:** Morbidity, Hospital records, Adolescent health, Morbidade, Registros hospitalares, Saúde do adolescente

## Abstract

**Objective:**

To describe hospital admissions of adolescents living in Sergipe, Northeast Brazil, from 2002 to 2012.

**Methods:**

Descriptive study, based on data collected from the Hospital Information System of the Unified Health System. Hospital admissions were divided into four groups of causes: by pregnancy, childbirth and puerperium; by external causes; by primary care conditions; and other causes. Numbers, percentages and coefficients were used in the analysis and compared by year, sex, age (from 10 to 14 and from 15 to 19 years), and the average annual cost of hospitalizations for each group of causes.

**Results:**

In the period studied, there were 149,850 hospital admissions of adolescents, 58.4% for pregnancy, childbirth and puerperium, 9.3% for primary care conditions, 8.3% for external causes and 24.0% for other causes. All coefficients decreased from 2002 to 2012 by 39.7%. Primary care conditions had the most significant reduction (143.1%), followed by external causes (60.1%). As for age groups, the coefficients for external causes in the age group of 15 to -19 years, and for pregnancy, childbirth and puerperium, in the age range of 10 to 14 years, are noteworthy because they remained stable in the period. There was an increase in the average cost of all admissions (234.7%), especially for external causes.

**Conclusions:**

Health actions to reduce hospital admission due to conditions sensitive to primary care should be given more attention, as well as those related to external causes and pregnancy, among adolescents living in Sergipe, Northeastern Brazil.

## RESUMO

**Objetivo:**

Descrever as internações hospitalares de adolescentes residentes em Sergipe, de 2002 a 2012.

**Métodos:**

Estudo descritivo, a partir de dados do Sistema de Informação Hospitalar do Sistema Único de Saúde, no qual as internações foram divididas em quatro grupos de causas: primeiro, por gravidez, parto e puerpério; segundo, por causas externas; terceiro, por condições sensíveis à atenção primária; e quarto, demais internações. Para a análise, foram utilizados os números, percentuais e coeficientes, por ano, sexo, idade (de 10 a 14; e de 15 a 19 anos) e custo médio anual das internações segundo os grupos de causas.

**Resultados:**

No período, ocorreram 149.850 hospitalizações de adolescentes, sendo 58,4% por gravidez, parto e puerpério; 9,3% por condições sensíveis à atenção primária; 8,3% por causas externas; e 24,0% foram as demais internações. Houve redução de 39,7% em todos os coeficientes entre 2002 e 2012, sendo que as por condições sensíveis à atenção primária apresentaram maior redução (143,1%), seguidas das causas externas (60,1%). Em relação às faixas etárias, chama a atenção os coeficientes por causas externas na idade de 15 a 19 anos e a gravidez, parto e o puerpério, dos 10 a 14 anos, por apresentarem estabilidade no período. Houve aumento do custo médio de todas as internações, de 234,7%, destacando-se o das causas externas.

**Conclusões:**

Impõe-se a necessidade de se incrementar ações de saúde para diminuir as hospitalizações por condições sensíveis à atenção primária, à incidência das causas externas e à gravidez entre os adolescentes sergipanos.

## INTRODUCTION

Knowing the morbidity and mortality profile of adolescents is relevant so one can come up with feasible prevention measures in primary care, allowing oriented actions and health services. Thus, the allocation of financial resources and the setting up of hospital structures for adequate secondary and tertiary care permeates this prior knowledge.^1^

Among the problems reported in adolescence, pregnancy, often unwanted, is a major concern which causes health, education, economic and social-development problems, causing higher school dropout rate, family problems and difficulties to enter the labor market.^2^ Therefore, pregnant adolescents are at higher biopsychosocial risk, especially those from lower social classes, as they end up facing even more difficulties in schooling and professionalization. In addition, an unwanted pregnancy at this age group naturally leads to greater biological risks for both the mother and the newborn.^3^

However, of all morbidity causes that affect adolescents, external causes are the most visible, as they cause death or permanent disability,^4^ being more frequent among adolescents aged between 15 and 19 years.^5^ ^,^ ^6^ Hospital admissions due to conditions sensitive to primary care, on the other hand, have been used as an indicator to evaluate primary care programs,^7^ especially in studies that aim to verify to what extent these programs, as they reach their goals, improve the health status of populations.^8^ Therefore, in communities with high rates of hospitalizations related to this group of causes, one might suspect that access to primary care is still poor.^9^

And, finally, there are hospital admissions by “other causes”, often poorly managed, such as hernias, appendicitis or malignant neoplasms, which require medium and/or high complexity structures.^10^ In this context, the objective of this study was to describe hospital admissions of adolescents living in Sergipe, from 2002 to 2012, according to the four above-mentioned groups of causes.

## METHOD

Descriptive study conducted in 2011 in the State of Sergipe, Northeast Brazil, with 2,068,017 inhabitants,^11^ of which 19.8% were adolescents, and which ranks the twentieth among the 27 federation units per the Human Development Index^12^, with 9.0% of adolescents having health insurance.^13^ Based on the Hospital Information System of the Unified Health System (SIH-SUS),^14^ the causes for hospital admission were divided into four groups, namely: pregnancy, childbirth and puerperium (PCP); external causes (EC); conditions sensitive to primary care (CSPC); and other causes (OC). To this end, the tenth revision of the World Health Organization’s International Statistical Classification of Diseases and Related Health Problems (ICD-10r) was applied.^15^ In the case of PCP admissions, in addition to the codes of the chapter on pregnancy, childbirth and puerperium (chapter XV), those of chapter XXI were used, that is, referring to search for health services due to circumstances related to reproduction^16^ (between Z31 and Z39), as determined by the Ministry of Health.

For EC, there is a decree by the Ministry of Health^17^ indicating that, for secondary diagnosis, the code of the external cause motivating hospital admission must be inserted (chapter XX of ICD-10), as it states the circumstance of injury. This was the diagnosis criteria used in the present study. The list provided by the Ministry of Health was used for CSPC^18^ and, finally, OCs were composed of all the remaining codes. Population data were obtained from SUS’ Department of Informatics (DATASUS).^11^

The cases were described according to numbers, percentages and coefficients for the four groups of causes. The variables were: year of admission, gender, age (from 10 to 14 and from 15 to 19 years), main diagnoses upon hospitalization (in two periods: 2002 to 2004 and 2010 to 2012), and mean cost (in dollars). Hospitalization’s values in dollars (listed in SIH-SUS)^19^ were divided by the number of admissions each year, for the four groups of causes, and the mean annual cost was then calculated. This study was approved by the Research Ethics Committee of Universidade Federal de Sergipe (CAEE No. 32811014.5.0000.5546).

## RESULTS

**Figure 1:**
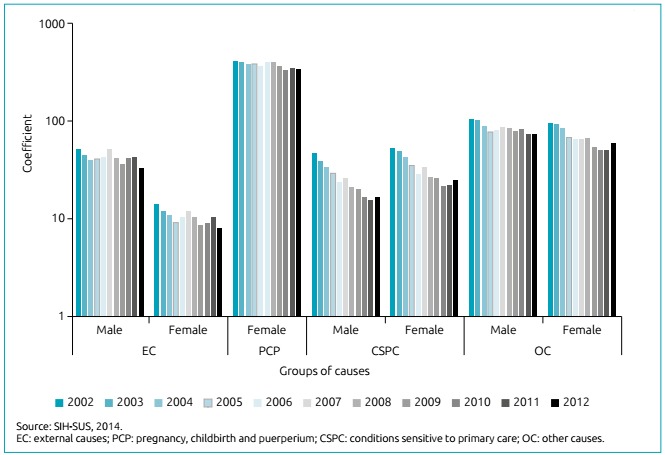
Hospital morbidity coefficient among adolescents due to external causes; pregnancy, childbirth and puerperium; conditions sensitive to primary care; and other causes according to gender. Sergipe, 2002 to 2012.

Over the study period, 149,850 hospital admissions of adolescents took place, 58.4% of them by PCP; 9.3% by CSPC; 8.3% by EC; and 24.0% by OC. Figure 1 shows the coefficients for each condition according to gender, in which PCP for females and OC for males stood out. There was a reduction of all coefficients in the period, on average by 39.7%; CSPC was shown to have the largest reduction (143.1%), followed by EC (60.1%), OC (49.1%) and PCP (21.1%).

**Figure 2:**
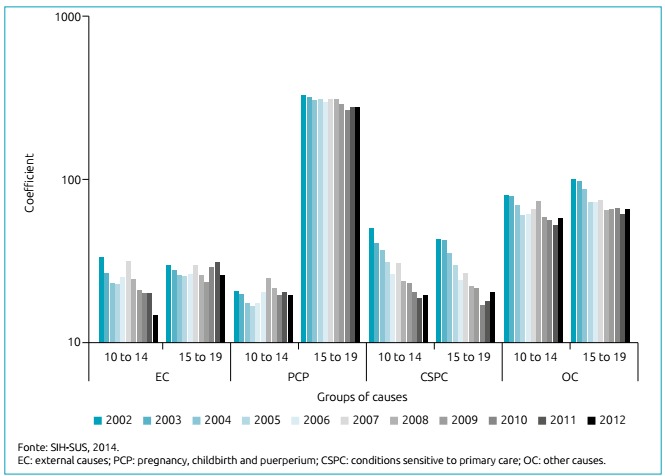
Hospital morbidity coefficient among adolescents due to external causes; pregnancy, childbirth and puerperium; conditions sensitive to primary care; and other causes according age group. Sergipe, 2002 to 2012.

Regarding age versus cause (Figure 2), the morbidity coefficients for PCP and OC in the age group 15-19 years were the highest. The following had a stability trend in the period: EC in the age group of 15 to 19 years and PCP in the range of 10 to 14 years old. The other groups showed a downward trend, the most marked being those related to CSPC and OC in both age groups.

Table 1 shows, for each group of causes (EC, PCP, CSPC and OC), the main diagnoses in numbers and percentages, according to the two study periods (2002 to 2004 and 2010 to 2012).

**Table 1:** Distribution of the main hospitalization diagnoses among adolescents, in number and percentage, according to four groups of causes. Sergipe, 2002 to 2004 and 2010 to 2012.

Groups of causes	Period			
	2002 to 2004		2010 to 2012	
	n	%	n	%
External causes*				
Falls (W00-W19)	2,542	67.0	1,591	52.0
Events (facts) of undetermined intention (Y10-Y34)	534	14.1	862	28.2
Transport Accidents (V01-V99)	275	7.2	335	11.0
Assault (X92-Y08)	93	2.5	123	4.0
Exposure to Smoke, Fire and Flames (X00-X09)	103	2.7	76	2.5
Other external causes	248	6.5	70	2.3
Subtotal	3,795	100.0	3,057	100.0
Pregnancy, childbirth and puerperium				
Childbirth (O80-O84)	20,727	79.3	16,035	75.1
Pregnancy ending in abortion (O00-O08)	3,033	11.6	1,809	8.5
Complications of labor and childbirth (O60-O75)	1,423	5.4	1,782	8.3
Assistance to the mother, amniotic cavity, birth problems (O30-O48)	451	1.7	1,146	5.4
PCP-related edema, proteinuria and hypertensive disorders (O10-O16)	214	0.8	234	1.1
Other causes related to PCP	304	1.2	354	1.7
Subtotal	26,152	100.0	21,360	100.0
Conditions sensitive to primary care				
Infectious intestinal diseases (A00-A09)	1,490	25.9	472	19.2
Influenza and pneumonia (J09-J18)	1,344	23.3	479	19.5
Chronic Lower Airway Diseases (J40-J47)	929	16.1	296	12.1
Tubulointerstitial Kidney Diseases (N10-N16)	522	9.1	81	3.3
Metabolic disorders (E70-E90)	282	4.9	60	2.4
Other causes related to CSPC	1,196	20.8	1,065	43.4
Subtotal	5,763	100.0	2,453	100.0
Other causes				
Herniations (K40-K46)	1,041	8.5	661	8.0
Health services, procedures and specific care (Z40-Z54)	484	4.0	518	6.3
Appendix Diseases (K35-K38)	428	3.5	656	8.0
Malignant Neoplasms (C00-C97)	590	4.8	497	6.0
Other bacterial diseases (A30-A49)	811	6.6	327	4.0
Other causes	9,035	72.5	5,570	67.7
Subtotal	12,389	100.0	8,229	100.0

Source: SIH-SUS, 2014.

*For external causes, the secondary diagnosis after hospital admission was used; PCP: pregnancy, childbirth and puerperium; CSPC: conditions sensitive to primary care.

**Figure 3:**
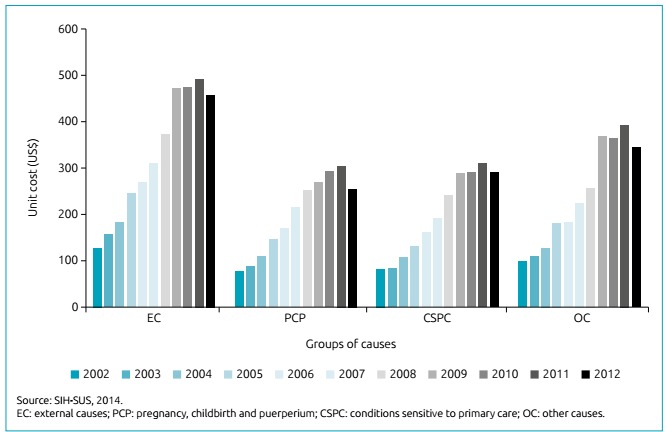
Hospital admissions of adolescents by external causes; pregnancy, childbirth and the puerperium; conditions sensitive to primary care; and other causes, according to costs of hospitalization. Sergipe, 2002 to 2012.

Figure 3 shows the mean costs for the groups analyzed, notably PCP, which, despite representing the lowest value, accounted for more than half of all hospitalizations in the period. EC, on the other hand, represented the highest value in the period. Such costs increased from US$ 127.66 to US$ 457.99 for EC; and from US$ 77.54 to US$ 254.75 for PCP between 2002 and 2012. The total cost of hospitalizations increased by 234.7% in the period.

## DISCUSSION

In this study, hospitalizations by PCP, especially at the age of 15-19 years, and EC among males stood out. From 2010 to 2012, the main diagnoses upon hospital admissions were: falls for EC, childbirth for CPC, influenza and pneumonia for CSAP and for hernias for OC. The four groups of causes showed an increase in the mean cost of hospitalizations from 2002 to 2012, especially EC.

For EC, important factors of disability and sequelae are seen, but which present themselves as a preventable problem.^6^ In Sergipe, there is a trend to stability in the age group of 15 to 19 years, while one sees a downwards trend for EC in the age group 10-14 years. This study corroborates the findings from the Clinical Hospital of the Medical School of Universidade de São Paulo, in Ribeirão Preto,^6^ where there was a continuous reduction in the number of visits between 2000 and 2006, as there was an improvement in resolution of cases in primary care and a greater reach of prevention actions.

Male ECs also predominate in Sergipe, as well as in Ipatinga, Minas Gerais, where 71.6% of EC admissions were of males and the most affected age group was 15-19 years.^5^ These results are also compatible with findings from a university hospital in the countryside of the state of São Paulo, where males accounted for 67.1% of EC visits and the age range between 15 and 19 years was the most affected.^6^

As for the main diagnosis of EC, falls accounted for 52.0% in Sergipe, followed by events of undetermined intent (28.2%), and transport/traffic accidents, representing 11.0% of cases from 2010 to 2012. This differs from a study at the Márcio Cunha Hospital, in Ipatinga, Minas Gerais, conducted between June and December 2009,^5^ which found the main EC to be transport accidents (37.7%), followed by falls (32.8%); and the findings at the Clinical Hospital of the Medical School of Universidade de São Paulo, Ribeirão Preto,^6^ in which the highest number of diagnoses was also related to traffic accidents (83.0%), but followed by assaults (13.0%).

Between 2002 and 2012, 58.4% of hospital admissions of adolescents in Sergipe were caused by conditions inherent to PCP. This percentage is similar to what other authors reported in studies conducted in the Southeast of Brazil, even in other temporal contexts. In the city of Rio de Janeiro, PCP represented 63.0% of the total admissions between 1993 and 1998.^20^ In Ribeirão Preto, São Paulo, in 1988, 1997 and 2006, it stood out as the main group of causes in the age range of 15 to 19 years.^1^ This high percentage highlights the need for more consistent interventions aimed at adolescent sexual and reproductive health,^3^ since the vast majority of adolescents know at least one contraceptive method, but still face difficulties in acquiring or using them correctly.^21^

The age group of 15 to 19 years old was the one that most used hospital services in Sergipe in the period, with emphasis to causes related to PCP and OC. This result is similar to what was found in a study conducted in Governador Valadares, Minas Gerais, between 2008 and 2011, where PCP was responsible for 70.6% of admissions of adolescents, followed by poisoning injuries (10.0%) in the same age group.^22^

PCP accounted for more than half of the total number of hospital admissions, and most adolescents were aged between 15 and 19 years, as in other studies conducted with adolescent mothers, in a maternity hospital in the city of São Carlos, São Paulo, from 2008 to 2009.^21^ In a historical series between 1970 and 2000,^23^ at the Vila Lobato Community Social Medical Center in Ribeirão Preto, São Paulo, adolescent mothers had mean age of 18 years. Attention should be given to this situation because of all the behavioral, financial and structural consequences that an unwanted pregnancy entails for the adolescent and her relatives.^23^

From 2010 to 2012, the main cause of hospital admissions related to PCP in Sergipe was childbirth (75.1%), followed by abortion (8.5%) and complications of labor and childbirth (8.3%). In Brazil, childbirth (68.7%) was the most common in 2012, followed by complications of labor and childbirth (8.8%) and assistance to the mother associated to the fetus, amniotic cavity and problems in childbirth (8,6%).^14^

Hospital admissions by CSPC had a reduction of 134.9% for both genders, considering both periods studied (2002 to 2014 and 2010 to 2012), which makes it the studied group of causes with the largest drop in these age groups. This information is important, as high rates of hospitalizations for CSPC may indicate deficiencies in access to the care system by the population of a given region, either due to poor service coverage or low rate of problem resolution in primary health care.^9^

In this study, infectious intestinal diseases were the main cause of CSPC in the first period (2002 to 2004) and pneumonia in the second period (2010 to 2012). These findings in the state of Sergipe partially coincide with results found in Brazil between 1999 and 2006, when gastroenteritis was the main cause in the age groups of 10 to 14 years and 15 to 19 years, with a downwards trend due to the improvement of therapeutic measures and the expansion of basic sanitation. Pneumonia was the third leading cause of hospital admissions under the age of 20 in Brazil between 1999 and 2006. Unlike Sergipe, which had a significant fall in its coefficients, the country, overall, tended to increase.^7^

In the OC group, hernias, appendix diseases and malignant neoplasms stood out, representing 16.6 and 22.0% of cases, respectively, in the first and second periods. With regard to hernias and appendix diseases, these represented 6.0% of adolescents enrolled in SIH-SUS in 1999, in Salvador, Bahia, corroborating the results of our study, as these causes are considered as inevitable hospitalization.^10^ Neoplasms have been the second leading cause of death between the ages of 5 and 19 in the country since 2004^24^ and, overall, children and youth tumors are more aggressive, although they respond better to chemotherapy.^25^ Still regarding malignant neoplasms, these alone represented 1,2 and 1.4% of cases from 2002 to 2004 and 2010 to 2012, respectively, similarly to the findings of the metropolitan region of Porto Alegre, Rio Grande do Sul, from 2008 to 2010, when these represented 1.0% of cases,^25^ but falls below the percentage presented in Salvador, Bahia, which was 3.0% in 1999.^10^

Regarding costs, in our study, hospital admissions due to EC had the highest mean values, followed by OC and the CSPC consequences. In Salvador, Bahia, in 1999, the mean expenditure per EC was also the highest. The costs per PCP, despite being the lowest mean value, corresponded to 58.4% of authorizations to admissions of adolescent.^10^ The differences in the mean values by group of causes may be justified by the higher cost of hospital components, such as diagnostic and intensive care methods, longer hospital stay for hospitalizations resulting from accidents or violence.

One of the limitations of this study is noteworthy that it is a descriptive study based on secondary data provided by the National Health Information system and, in this sense, and therefore subject to biases related to data quality. However, a survey on the scientific production of SIH-SUS data applications indicated that, despite the system having incomplete coverage and uncertain information reliability, it has shown internal consistency and consistency with current knowledge, which reinforces its importance and potential of use.^26^ Another limitation is that the data refer only to hospital admissions financed by the Unified Health System, but, as mentioned, they make up 90.0% of the total. Finally, one must be cautious not to generalize these data to other regions of the country, as there are possible ethnic, cultural and socioeconomic differences.

This study showed that knowing the morbidity and mortality profile that affects adolescents in a given region enables authorities to implement effective health prevention measures, actions and services to reduce these coefficients, since in most cases the demand for hospital services by adolescents takes place in urgency and emergency services.^20^ These actions should not be isolated, and should have the whole society participating, through municipal and local health councils, as well as policies involving other sectors such as sports, leisure and education.^22^ In addition, other studies have also shown that there are still high rates of inadequate demand by children and adolescents in hospital units, being considered inadequate the diagnoses related to patients that could have been assisted in primary care.^27^ This is what a survey conducted in Maceió, Alagoas,^28^ shows: 83.2% of inadequacy. Another study in Recife, Pernambuco,^27^ reported only 25.5. % of suitability for all ages. Thus, in Sergipe, health actions need to be extended to reduce hospital admissions for conditions sensitive to primary care and the incidence of external causes and pregnancy

## References

[1] Del Ciampo LA, Del Ciampo IR (2011). Morbidity profile and hospitalization of adolescents at Ribeirão Preto (SP) region. Medicina (Ribeirão Preto).

[2] Manfré CC, Queiróz SG, Matthes AC (2010). Considerações atuais sobre gravidez na adolescência. Rev Bras Med Fam Comunidade.

[3] Dias AC, Teixeira MA (2010). Gravidez na adolescência: um olhar sobre um fenômeno complexo. Paidéia (Ribeirão Preto).

[4] Waiselfisz JJ (2012). Mapa da Violência 2012: crianças e adolescentes do Brasil.

[5] Gaspar VL, Souza EC, Carmo JH, Pereira WD (2012). Características de crianças e adolescentes hospitalizados em decorrência de causas externas. Rev Assoc Med Minas Gerais.

[6] Silva MA, Pan R, Melo L, Bortoli PS, Nascimento LC (2010). Perfil dos atendimentos a crianças e adolescentes vítimas de causas externas de morbimortalidade, 2000-2006. Rev Gaúcha Enferm.

[7] Moura BL, Cunha RC, Aquino R, Medina MG, Mota EL, Macinko J (2010). Principais causas de internação por condições sensíveis à atenção primária no Brasil: uma análise por faixa etária e região. Rev Bras Saude Matern Infant.

[8] Starfield B (2002). Atenção primária: equilíbrio entre necessidades de saúde, serviços e tecnologia.

[9] Alfradique ME, Bonolo PD, Dourado I, Lima-Costa MF, Macinko J, Mendonça CS (2009). Internações por condições sensíveis à atenção primária: a construção da lista brasileira como ferramenta para medir o desempenho do sistema de saúde (Projeto ICSAP - Brasil). Cad. Saúde Pública.

[10] Nascimento EM, Mota E, Costa MC (2003). Custos das internações de adolescentes em unidades da rede hospitalar integrada ao SUS em Salvador, Bahia. Epidemiol Serv Saúde.

[11] Brazil - Ministério da Saúde (2014). DATASUS. Informações em saúde. Demográficas e socioeconômicas.

[12] Programa das Nações Unidas para o Desenvolvimento (PNUD) Atlas do desenvolvimento humano no Brasil.

[13] Brazil - Ministério da Saúde (2018). Informações em Saúde. Saúde Suplementar.

[14] Brazil - Ministério da Saúde (2014). DATASUS. Transferências de arquivos. Arquivos de dados. SIHSUS.

[15] Organização Mundial da Saúde (1995). Classificação estatística internacional de doenças e problemas relacionados à saúde - CID 10.

[16] Brazil - Ministério da Saúde (2014). DATASUS. Informações em saúde. Epidemiológicas e de Morbidade. Nota Técnica.

[17] Brazil - Ministério da Saúde. Secretaria de Assistência à Saúde (1997). Portaria nº 142, de 13 de novembro de 1997. Dispõe sobre o preenchimento de Autorização de Internação Hospitalar - AIH - em casos com quadro compatível com causas externas. Seção 1.

[18] Brazil - Ministério da Saúde (2008). Portaria GM/MS nº 221 de 17 de abril de 2008. Publica a lista brasileira de internações por condições sensíveis à atenção primária. Seção 1:70.

[19] Brazil - Ministério da Saúde Secretaria de Gestão Estratégica e Participativa. Divisão de Disseminação de Informações em Saúde. Disseminação de Informações do Sistema de Informações Hospitalares (SIH) Informe Técnico referente ao processamento 2016-03.

[20] Ruzany MH, Travassos C (2006). Internação hospitalar de adolescentes no município do Rio de Janeiro: como prevenir?. Adolesc Saúde.

[21] Beretta MI, Clápis CV, Oliveira LA, Freitas MA, Dupas G, Ruggiero SE (2011). A contextualização da gravidez na adolescência em uma maternidade de São Carlos/SP. Rev Eletr Enf.

[22] Santos RC (2015). Jovens, negros, mineiros e valadarenses: subjetividades marcadas pela vulnerabilidade social e violências.

[23] Ciampo LA, Junqueira MJ, Ricco RG, Daneluzzi JC, Ferraz IS, Martinelli CE (2004). Tendência secular da gravidez na adolescência. Pediatria (São Paulo).

[24] Grabois MF (2011). O acesso à assistência oncológica infantil no Brasil.

[25] Stüker UA (2013). Internações pelo SUS de crianças e adolescentes, por câncer, residentes na região metropolitana de Porto Alegre, RS, de 2008 a 2010 [specialization work].

[26] Bittencourt SA, Camacho LAB, Leal MC (2006). O Sistema de Informação Hospitalar e sua aplicação na saúde coletiva. Cad Saúde Pública.

[27] Furtado BM, Araújo JL, Cavalcanti P (2004). O perfil da emergência do Hospital da Restauração: uma análise dos possíveis impactos após a municipalização dos serviços de saúde. Rev Bras Epidemiol.

[28] Simons DA, Monlleó IL, Simons SA, Araújo JL (2010). Adequação da demanda de crianças e adolescentes atendidos na Unidade de Emergência em Maceió, Alagoas, Brasil. Rev Bras Saúde Mater Infant.

